# The Impact of the Implementation of International Law on Marine Environmental Protection on International Public Health Driven by Multi-Source Network Comment Mining

**DOI:** 10.3390/ijerph20065130

**Published:** 2023-03-14

**Authors:** Anqi Yang, Shudong Yang

**Affiliations:** School of Law, Chongqing University, Chongqing 400044, China

**Keywords:** international public safety, international law of marine environmental protection, greening urban spaces, green space quality, healthy community, PSO-K-means

## Abstract

With the increase of people’s living space, global warming caused by the decrease of greening urban spaces and the serious decline of greenspace quality has led to extreme weather events and coastal erosion, which has become the biggest threat to the ocean and has also led to the occurrence of international public safety incidents. Therefore, it is of great practical significance to explore the tense relationship between the current marine environmental protection and global public safety for the development of an international healthy community. Firstly, this paper discusses the influence of implementing the international law of marine environmental protection on global public health after the reduction of green urban space and the decline of green space quality. Secondly, K-means and discrete particle swarm optimization algorithms are introduced and the particle swarm optimization-K-means clustering (PSO-K-means) algorithm is designed to screen and deal with the mapping relationship between latent variables and word sets about the impact of implementing the international marine ecological protection law on the international public health community in network data information. Moreover, the influencing factors are clustered and the scenarios are evaluated. The results show that the clustering analysis of the marine environment can promote the clustering of marine characteristic words. Meanwhile, the PSO-K-means algorithm can effectively cluster vulnerability data information. When the threshold is 0.45, the estimated recall rate of the corresponding model is 88.75%. Therefore, the following measures have been formulated, that is, increasing greening urban spaces and enhancing the quality of green space to enhance the protection of marine environment, which has practical reference value for realizing the protection of marine environment and the sustainable development of marine water resources and land resources.

## 1. Introduction

In recent years, under the trend of the rapid expansion of people’s activity space, the green area of urban space has been seriously reduced and the quality of green space has decreased significantly. The resulting global warming has even led to a dramatic deterioration of the marine environment. In the era of big data, online comments profoundly affect individuals, groups, societies, and countries [1,2]. Public opinion formed through online comments has also led people to pay attention to the impact of marine pollution on human survival and development. The public constantly reflects on the relationship between human beings and the environment through the news about the situation to gradually form a substantial public opinion pressure and supervision role [3]. People expect to find the reasons for the increase in environmental pollution and the frequent occurrence of pollution incidents to analyze how to prevent environmental pollution [4]. In addition, the local division of data can be realized through the clustering algorithm of unsupervised learning. After clustering the data set, the clusters are analyzed separately to obtain more detailed results. Cluster analysis can be used in the data preprocessing stage to focus on complex data structures with specific algorithms to standardize complex data structures. Therefore, applying intelligent analysis methods to the analysis of global warming and marine environmental degradation, timely and accurately grasping relevant online commentary, and formulating and implementing environmental protection laws and policies have become the focus of many scholars in related fields.

Agglomerative hierarchical clustering (AHC) has been applied to opinion analysis several times in recent years. The AHC method is generally a small to large method. It starts by dividing each piece of data into a complete cluster. Then, according to the similarity of the data, the nearest clusters are gradually merged into a cluster until all clusters have merged into one cluster or some established conditions have been reached [5]. The K-means algorithm, density-based spatial clustering of applications with noise algorithm, and ordering points to identify the clustering structure algorithm of the AHC algorithm are preferred algorithms in the analysis methods [6,7]. Among them, density-based clustering has good effect and fast clustering speed. For each attribute divided, a single scan determines the number of mesh cells and grid cells per object. The K-means clustering algorithm is the most commonly used of all clustering algorithms. After artificially setting the initial K value, K objects are randomly selected to represent the center of K clusters. Next, the distance of other objects from the K centers is calculated and assigned to the nearest cluster. Then, the K-means algorithm iteratively improves the intra-cluster variation, calculates a new mean for each cluster, and takes the updated mean as the new cluster center point. All objects are reassigned to continue to iterate until they stabilize. The clustering algorithm has attracted much attention from experts and scholars because of the simplicity of the algorithm presented in the application, its easy implementation, and the good results of the application [8]. Currently, cluster analysis methods are widely used in medical diagnoses, image processing, information retrieval, statistics, biology, and other fields [9,10,11,12].

Therefore, the classical K-means algorithm in the AHC analysis method is selected to combine with the particle swarm optimization (PSO) algorithm. The purpose of this paper is to explore the relationship between the current situation of marine environmental protection and international public health safety by digging up online comments on marine environmental protection. Its novelty lies in the design of the particle swarm optimization-K-means clustering (PSO-K-means) algorithm. Through the PSO-K-means algorithm, the mapping relationship between potential variables and word sets of marine protection status is clustered and the influencing factors are evaluated. In this way, the impact on global public health is explored by taking measures such as greening urban spaces, enhancing the quality of green spaces, and implementing the international marine environmental protection law. In addition, the development trend of online public opinion is predicted, which provides a reference for formulating and implementing marine ecological protection policies.

## 2. Literature Review

At present, there are studies on global warming and the governance of the marine environment. Benedetti et al. (2021) found that zooplankton richness was expected to decline slightly in the tropics but to increase significantly in temperate to subpolar regions. Meanwhile, climate change threatened the contribution of plankton communities to plankton-mediated ecosystem services such as biocarbon sequestration [13]. Bache and Reynolds (2022) reviewed ocean goals as part of the sustainable development goals and considered the process of linkage thinking in more detail, particularly in relation to ocean-climate linkages. It was found that relying on SDG interaction analysis was risky because the results were inaccurate or could not adapt to rapid transformation or knowledge acquisition in some areas. They also recognized that planetary boundary tipping points would help bring the oceans into climate consideration [14]. Therefore, the strategies of marine environmental protection and climate management are analyzed through the literature collation of environmental protection and public health. It promotes the improvement of algorithm operation efficiency while saving storage space resources, which has practical reference significance for establishing international public security model.

As one of the data mining techniques, clustering algorithms can extract useful information from massive data. Numerous scholars have studied it. Bu et al. (2019) applied the classical K-means technique to model a realistic social media network as a discrete-time dynamic system. They performed social media information analysis regarding opinion matrix and community structure interactions [15]. Tang (2022) used the K-means algorithm for mining and analyzing the public opinion hotspots of Chinese microblogs, which could grasp the public opinion hotspots easily and quickly after experiments [16]. Rahim Taleqani et al. (2019) performed data analysis using cluster analysis on 32,000 comments on Twitter on the issue of bicycle sharing, which precisely derived from people’s concerns about bicycle sharing [17]. K-means clustering analysis and data mining techniques based on K-means clustering analysis can also be used in finance. Li et al. (2020) used K-means clustering analysis to analyze the investment efficiency of smart investments and had good results [18]. The losses of major enterprises during the new coronavirus can also be explored in the data. Zheng et al. (2022) analyzed the deviation from the state support policy for enterprises in southern China, thus providing ideas for their continued development [19]. The results of the literature review data are summarized in Table 1.

Through the research of the above scholars, it is found that, under the global warming trend, people have paid attention to urban space greening and marine environmental governance. Moreover, cluster analysis methods have become indispensable in the era of big data and cluster analysis methods will greatly save human and material resources. However, applying clustering algorithms to marine environmental analysis is extremely rare. Therefore, this paper improves the cluster analysis algorithm and applies it to the analysis of the marine environment and the formulation of countermeasures, which can provide a reference for the subsequent green and sustainable development of the marine environment.

## 3. Methods and Models

### 3.1. K-Means Clustering Algorithm

**Figure 1:**
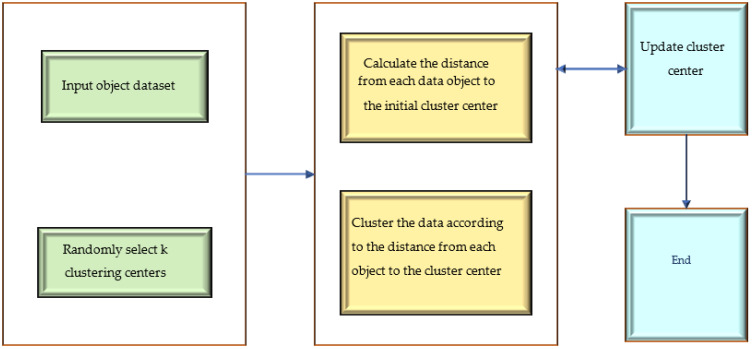
K-means algorithm flow.

K-means is a partitional clustering algorithm called the K-means algorithm; it has unique advantages in extensive data analysis and information mining [20]. Figure 1 shows the workflow of the K-means clustering algorithm.

In Figure 1, K-means starts from K randomly selected centroids to define the prototype of the cluster structure. Each data point is assigned to the cluster to which the nearest centroid belongs according to the distance. Then, the centroid position of each cluster is updated iteratively (the new centroid is usually the mean of the data points within the cluster and it hardly corresponds to the actual data points) until the positions of all the particles no longer change. The K-means algorithm is efficient and scalable when dealing with datasets. The optimal state is where the algorithm’s K division squared error values tend to be the smallest. The effect is good when the distribution characteristics of the clustering results are apparent. The disadvantage of the K-means algorithm is that it can only be used if the cluster’s mean is pre-defined. Therefore, choosing a suitable initial centroid is a critical step in the K-means algorithm.

### 3.2. Discrete Particle Swarm Optimization (DPSO) Algorithm

The PSO algorithm is proposed for the continuous function optimization problems. The algorithm is built on a constant domain to optimize the solution [21]. A PSO algorithm suitable for the discrete binary version is proposed after modifying the basic PSO to solve the discrete optimization problem. In the DPSO algorithm, the value of each dimension of the particle’s position vector *t* is limited to integer zero or one. There is no limit to the particle’s velocity, but velocity is usually used to characterize the probability that each dimension in the position vector takes an integer zero or one. If the velocity value *v* of a particular dimension *j* of particle *i* is larger, the probability that the corresponding position of the particle takes an integer one is higher. Otherwise, the probability of taking zero is greater. The sigmoid function conforms to this characteristic, so the sigmoid function is used in the PSO algorithm to convert the range of the speed *v*. The function conversion method is as follows.

(1) $$sigmoid\left(v_{i}^{j}(t+1)\right)=\frac{1}{1+e^{-v_{i}^{j}(t+1)}}$$

The sigmoid function increases monotonically on the interval (+∞,−∞). It has a value of 0.5 at coordinate *r* = 0. The equation of the discrete binary PSO algorithm based on the above sigmoid function is as follows. In addition to the particle’s moving speed needing to be further converted, its basic speed equation and the standard particle swarm algorithm’s speed equation are as follows.

(2) $$v_{i}^{j}(t+1)=v_{i}^{j}(t)+\left[c_{1}r_{1}(t_{i}^{j}-x_{i}^{j}t)\right]+\left[c_{1}r_{2}(t_{i}^{j}-x_{i}^{j}t)\right]\;\;\;\;\;\;\;\;\;\;\;\;\;\;\;\;\;\;\;\;\;\;\;\;\;\;\;\;\;\;\;\;\;\;\;\;\;$$

(3) $$v_{i}^{j}(t+1)=\;sigmoid\left(v_{i}^{j}(t+1)\right)\;\;\;\;$$

(4) $$v_{i}^{j}(t+1)=\left\{\begin{array}{c} 0,r_{i}^{j}(t)>v_{i}^{j}(t+1)\\ 1,r_{i}^{j}(t)\leq v_{i}^{j}(t+1) \end{array}\right\}\;\;\;\;\;$$

In Equations (2)–(4), $r_{i}^{j}(t)\sim \cup (0,1)$ represents a random value that follows a normal distribution. It is mainly used to limit the probability of the speed value. *c*_1_ is the initial center vector and *c*_2_ is the largest center vector. In addition, the maximum speed limit is also preserved in the PSO algorithm. The greater the value of $\left|v_{i}^{j}(t)\right|<V_{MAX}$, the greater the corresponding probability value and the greater the variation probability of the particle dimension value. From the curve of the sigmoid function $f\left(x\right)=\frac{1}{1+e^{-v_{i}^{j}\left(t+1\right)}}$, the value of the sigmoid function tends to zero and $x_{i}^{j}=0$ when the *j*th dimension velocity of particle *i* is $v_{i}^{j}<10$. Similarly, the value of the sigmoid function tends to one and $x_{i}^{j}=1$ when the particle’s velocity $v_{i}^{j}>10$. Therefore, it is necessary to limit the maximum speed range to prevent the algorithm from being stagnant in the search in the process of binary encoding.

### 3.3. PSO-K-Means Algorithm

**Figure 2:**
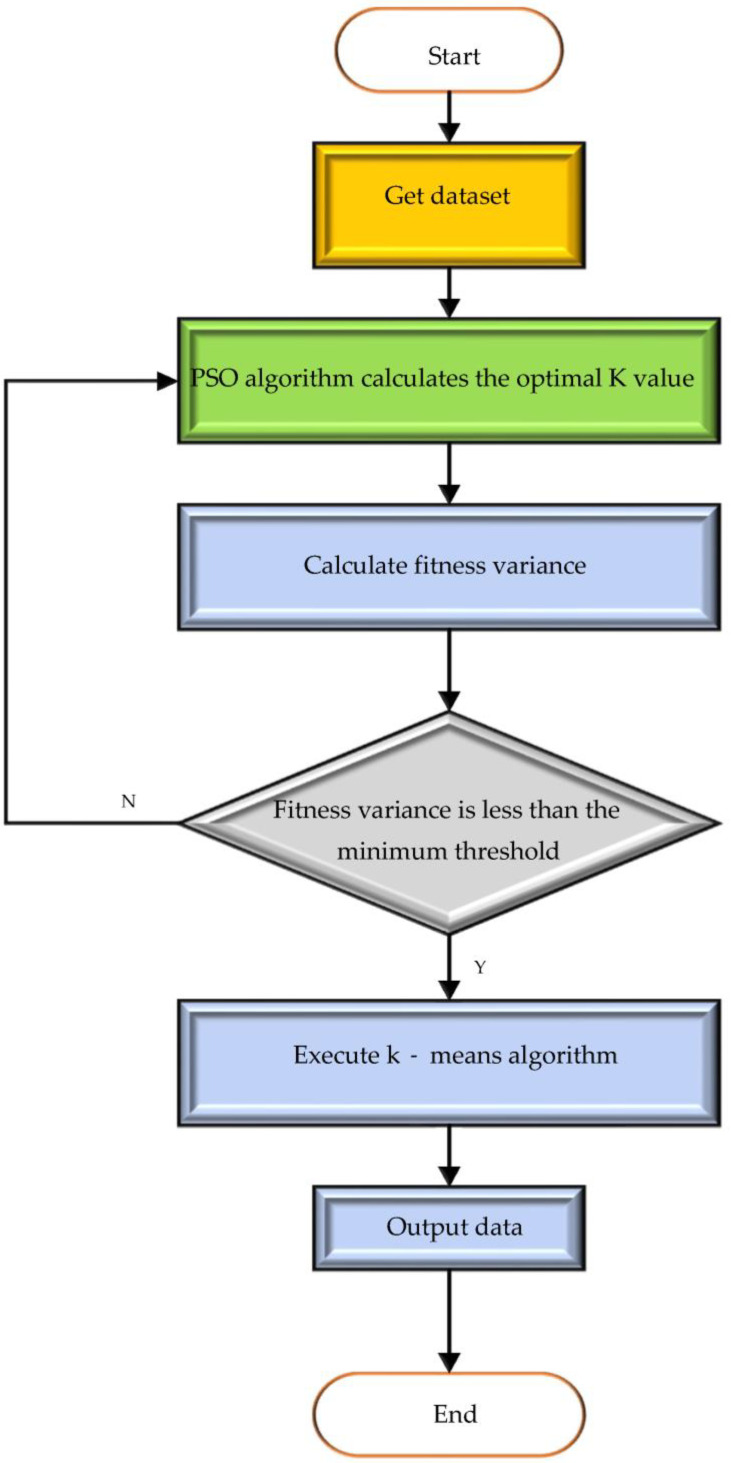
PSO-K-means algorithm flow.

The K-means clustering algorithm is considered one of the most influential and popular data mining algorithms among the AHC algorithms. Despite its popularity, this algorithm has certain limitations, including issues related to the random initialization of the centroids. This can lead to unexpected convergence. Therefore, this paper chooses to use the PSO algorithm to determine the initial centroids of the K-means clustering algorithm. In this way, the K-means clustering algorithm can select the optimal K value in the marine environmental protection comment analysis. The optimal analysis results can also be obtained. Here, the combination of the two algorithms is called the PSO-K-means algorithm. From Figure 2, the number of clusters K is obtained based on the optimal solution obtained by the PSO algorithm. K-means finds the cluster structure represented by K centroids.

Applying the PSO algorithm to the K-means algorithm can quickly search and accurately find the initial cluster center of the K-means algorithm. The function of evaluating the clustering effect is taken as the fitness function of the particle swarm. The frequent word space set $K_{sst}={\left(k_{s}^{1},k_{s}^{2},k_{s}^{3}\cdots k_{s}^{m}\right)}^{T}$ is divided into K categories. Then, the fitness function is:

(5) $$g\left(x\right)=\sqrt{\sum_{i=1}^{m}\sum_{j=1}^{k}{\left(k_{s}^{i}-c_{j}\right)}^{2}}\;\;\;\;\;\;\;\;\;$$

In Equation (5), *m* is the total number of particles in the PSO algorithm and *i* is the particle number. *k* is the initial number of particles in the K-means algorithm. *s* is the total number of texts. *c* is the cluster. *j* is the number of clusters.

The fitness variance can reflect the convergence degree of the particle swarm and the group fitness variance can be obtained according to Equation (6).

(6) $$\delta^{2}=\frac{1}{n}\sum_{i=1}^{n}\left[g(x)-\overset{-}{g}\right]\;\;\;\;\;\;\;\;\;\;\;\;\;\;\;\;\;\;\;\;\;\;\;\;\;\;\;\;\;\;\;\;\;\;\;\;\;\;\;\;\;\;\;\;\;\;\;\;\;\;\;\;\;\;\;\;\;\;\;\;\;\;\;\;\;\;\;\;\;\;\;\;\;\;\;\;\;\;\;\;\;$$

In Equation (6), *g* is the population’s average fitness. When $\delta^{2}$ is less than the set threshold, the fitness value fluctuates less. The particle swarm is in a convergent state. At this point, terminating the PSO algorithm and executing the K-means algorithm can make the later convergence speed fast.

**Figure 3:**
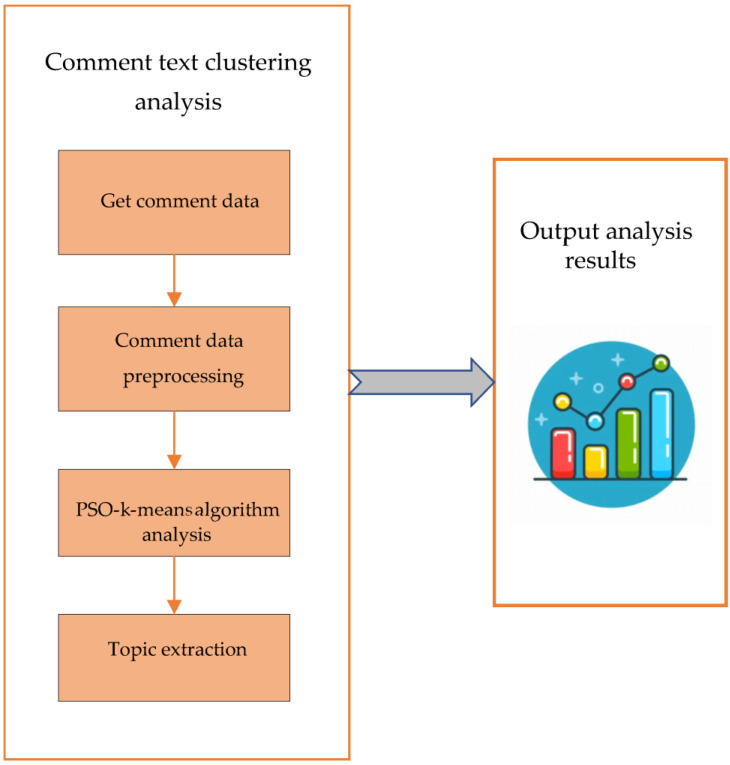
Application of PSO-K-means algorithm.

PSO-K-means is applied to multi-source network comment mining to explore the impact of the implementation of international law on marine environmental protection on global public health. From Figure 3, the review information text is collected first. The PSO-K-means algorithm is used for cluster analysis and topic extraction to obtain the analysis after data preprocessing.

### 3.4. Preprocessing of Online Comment Data

**Figure 4:**
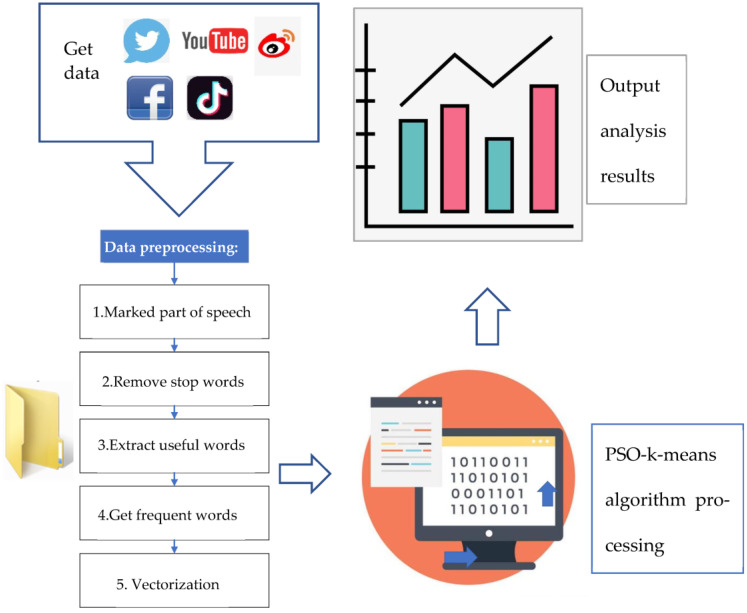
Analysis of the comments data of the marine environmental protection network.

From Figure 4, comment information mining needs to go through four processes: data collection, preprocessing, text clustering, and result in analysis. First, the data source of this analysis is the information related to marine environmental protection obtained from major social networking and news websites through crawler programs. After preliminary screening, a total of 5931 data texts are obtained and recorded as the original marine environmental protection review data set. Secondly, data preprocessing is performed to extract the information on the features of the online reviews and put them into the text. Finally, the text is preprocessed to obtain the feature vector, generating compelling information. In the process of text clustering, the PSO algorithm is used to find the cluster center intelligently. Then, clustering is implemented using the K-means algorithm. During the analysis of the clustering results, the marine environmental protection information after topic extraction is obtained by comparing the relevant high-frequency comments. The accuracy of the PSO-K-means clustering algorithm in clustering comment information is evaluated. Then, the algorithm’s performance is analyzed and compared with the pure K-means algorithm for accuracy convergence. Finally, the application of the algorithm in practice is evaluated.

The detailed steps of data preprocessing are as follows.

1. The first is to mark the part of speech. A tokenizer is used to tag high-frequency words in each comment.
2. The second is to remove stop words. Stop words are meaningless words that need to be removed. Remove meaningless words (such as am, is, are, and for).
3. The third is to extract valuable words. Proper nouns, verbs, and adjectives are extracted to ensure that the extracted words have actual meanings and can accurately express the characteristic information of comments.
4. The fourth is to obtain frequent words. All recorded frequencies are counted. High-frequency words have a representative role in this description field. Words that are representative and exceed the word frequency threshold are selected as representative elements combined with comment elements and high-frequency vocabulary.

A set of documents D(d1,d2,d3⋯dn) and a set of words W(w1,w2,w3⋯wn) are given. d represents a document and w represents a high-frequency word. Assuming that the order and position of each word in the text are ignored, a d-w matrix can be formed, as shown in Equation (7).

(7) $$A={\left[n(d_{i},w_{i})\right]}_{\left|D\right|\times \left|W\right|\;\;\;\;\;\;\;\;\;\;\;\;\;\;\;\;\;\;\;\;\;\;\;\;\;\;\;\;\;\;\;\;\;\;\;\;\;\;\;\;\;\;\;\;\;\;\;\;\;\;\;\;\;\;\;\;\;\;\;\;\;\;\;\;\;\;\;\;\;\;\;\;\;\;\;\;\;\;\;\;\;\;\;\;\;\;\;\;\;\;\;\;\;\;\;\;\;\;\;\;\;\;\;\;\;\;\;\;\;}$$

**Figure 5:**
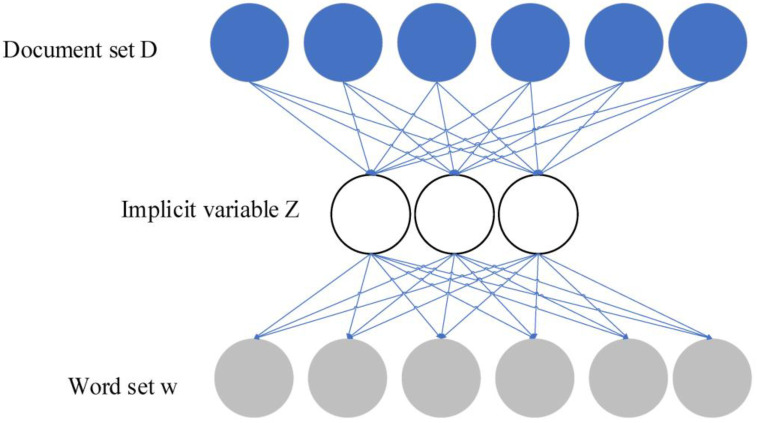
Mapping relationship among document set, latent variable, and word set.

In Equation (7), $n\left(d_{i},w_{i}\right)$ represents the word frequency of the word $w_{i}$ in the document $d_{i}$. In the latent variable set $Z(z_{1}z_{2}\cdots z_{n})$, *z* represents the latent variable that has not been observed. Therefore, *d* and *w* are independent of each other and *k* is based on experience. The correspondence between the three-level variables of “text-implicit-word” is shown in Figure 5.

5. The fifth is the vectorization of representative comment information. Through preprocessing, the dataset is segmented and stop words removed to turn it into a set of words. Each comment in the comment dataset is converted into a vector format of $\left(t_{1},w_{i1};t_{2},w_{i2}\cdots t_{n},w_{in}\right)$ to convert textual data into format vector data. $t_{n}$ refers to a word in a comment. The next is the weight value $w_{in}$ that the word has. All articles are integrated into vector format as input to the PSO-K-means algorithm.

Additionally, in the performance evaluation of model data mining, word frequency refers to the frequency of feature words in the text dataset. Recall refers to the proportion of data in the algorithm that is true positive and judged to be positive. The number of network comments for feature words can be judged by word frequency and the optimization performance of different algorithms can be evaluated through recall. Word frequency and recall can be obtained according to Equations (8) and (9).

(8) $$Word\;frequency=\frac{F1}{F2}$$

(9) $$\mathrm{Recall}=\frac{TP}{TP+FN}$$

In Equations (8) and (9), *F*1 represents the number of occurrences of a particular phrase in the text. *F*2 refers to the total number of independent phrases that divide the dataset text into pieces. *TP* represents the total number of samples that are actually positive and predicted to be positive. *FN* represents the total number of samples that are actually positive and predicted negative.

### 3.5. Clustering of Related Online Comment Texts

**Figure 6:**
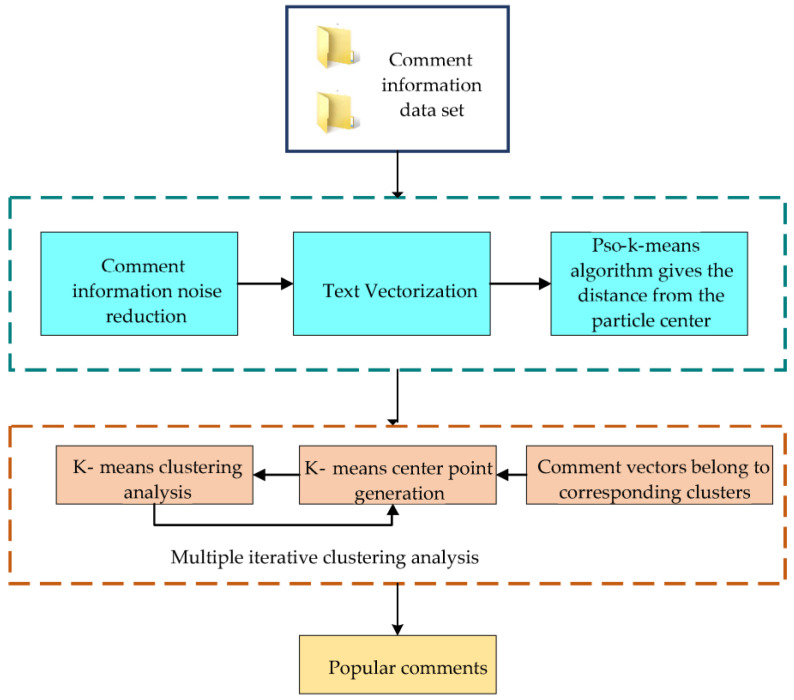
Clustering of online comment texts.

After processing the original marine environmental protection review data set obtained above, the relevant online review texts are initialized by the PSO algorithm, as shown in Figure 6. The remaining objects are divided into the nearest classes according to their distances from each cluster center and iterate continuously until the function converges. The original complex comment data set can be normalized and the comment hotspots from different aspects of the commenting object can be extracted from it. Then, the feature information extraction of online comments is realized to accurately grasp the hotspots and key points of the online comments.

The PSO-K-means clustering algorithm is used to obtain the distance of each vectorized information from the particle after the noise reduction and vectorization of the comment information. Then, cluster according to distance. Firstly, the comment vector with the largest local density is selected from the set as the initial center vector C1 of the maximum and minimum distance algorithm. The maximum and minimum distance algorithm can find the center point accurately combined with the reduced candidate vector set. The distance between other vectors in the dataset and C1 is calculated and the vector C2 with the largest distance is selected as the second center vector. The distance from each comment vector to each center vector in the dataset is calculated and denoted as *D_ij_*. The minimum values in *D_ij_* are selected to form the minimum distance from the sample to the center vector set. The largest distance *D_w_* in this set is chosen. In addition, the vector *C_w_* corresponding to *D_w_* as a new center vector is added to the center vector set to continue to search for the next center vector until the maximum *D_ij_* is satisfied. Meanwhile, *D_ij_* is assigned to *T*1. The purpose is to select the center of each comment vector as large as possible to avoid falling into the optimal local solution. Through the cluster analysis stage, multiple clusters can be obtained. Each cluster represents a hot spot in this review dataset. According to the corresponding comment vector in each hotspot, the word vector combination with the largest weight is extracted, representing the hotspot’s main text content. Sorting out the hotspot information in each cluster realizes the extraction of all hotspot information in this review dataset.

## 4. Results and Discussion

### 4.1. Cluster Analysis of Marine Environmental Reviews

Only 4830 of the original 5931 are left after denoising, deduplication, word segmentation, stop word filtering, and other text preprocessing operations on the original marine environmental protection review dataset. When performing text preprocessing operations, the evaluation command is first issued to the background by any user and the background system selects the appropriate data range through the functional logic layer. After the initial population data query is carried out through the data layer, the clustering calculation of each item is performed on the model range data. The data of the clustering model is retained before the judgment operation of the clustering model is performed. After the original text set is converted into structured data, term clustering and document clustering are performed on the data set in the cluster analysis process. For the document clustering process, the representative output term is set to three at a time. K-means cluster analysis is performed on structured data. The number of initial clusters is set to four, five, and six by the PSO algorithm. Different K values will have different effects on the clustering results. The clustering results using PSO-K-means are shown in Table 2.

**Figure 7:**
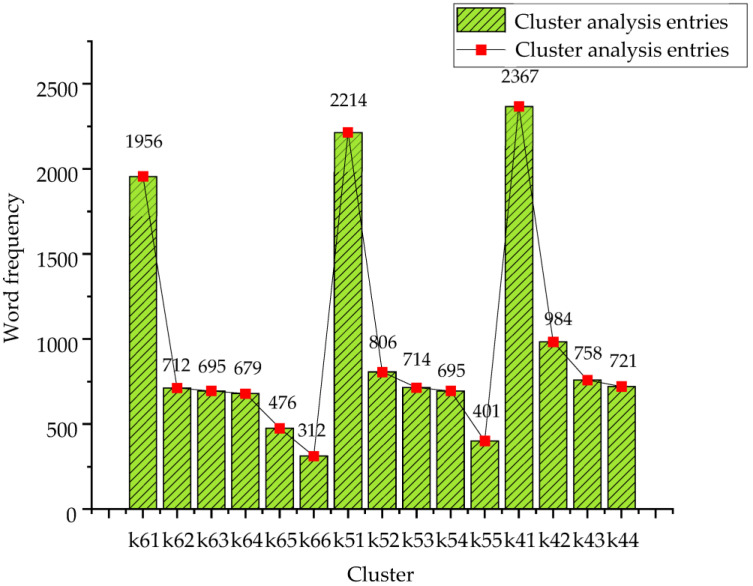
Cluster analysis diagram of marine environmental protection comments.

From Table 1, after clustering analysis of 4830 valid comments, different K values will output different clustering results. The less the K value is set, the fewer the classification results of the representative entries and the easier it is to obtain the analysis results. The larger the K value is set, the more detailed the classification will be and the more complex the analysis results will be. Furthermore, the clustering time and the number of iterations are also affected. After preprocessing the comment text and text representation, the feature items are counted. Figure 7 displays the cluster analysis graph.

**Figure 8:**
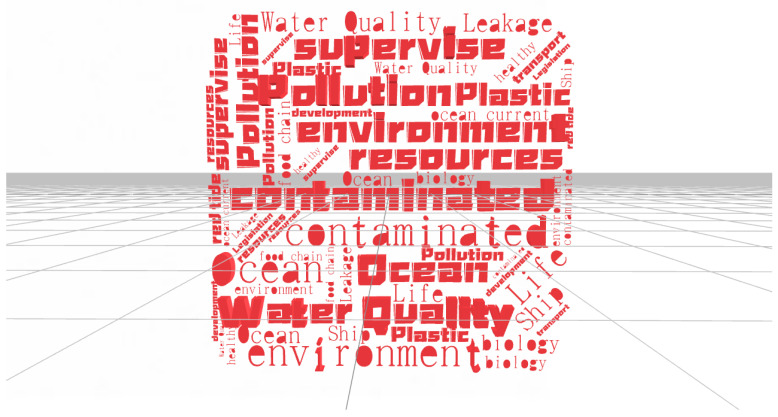
Schematic diagram of the feature word cloud.

For the form of feature item visualization, the word cloud diagram is shown in Figure 8. “Word cloud” is to visually highlight “keywords” that frequently appear in web texts by forming a “keyword cloud layer” or “keyword rendering”. There are more than 4000 feature items in the review set, so the feature items with a word frequency higher than 300 are used when drawing the word cloud and those with a word frequency lower than 300 are eliminated. When drawing the word cloud map, a sparsity of 0.98 is selected and 18 high-frequency feature items are retained to display the relationship between feature items.

From Figure 8, feature items with high frequency are clustered in the middle area of the word cloud; however, high frequency does not mean important. Although some feature items are not in the middle area of the word cloud, their importance to comments is more important than the feature items in the middle area. From the word cloud network constructed from 18 feature items, the words near the central region are large. This indicates that its importance is high and the connection between the feature terms is strong.

### 4.2. PSO-K-Means Algorithm Experimental Evaluation

**Figure 9:**
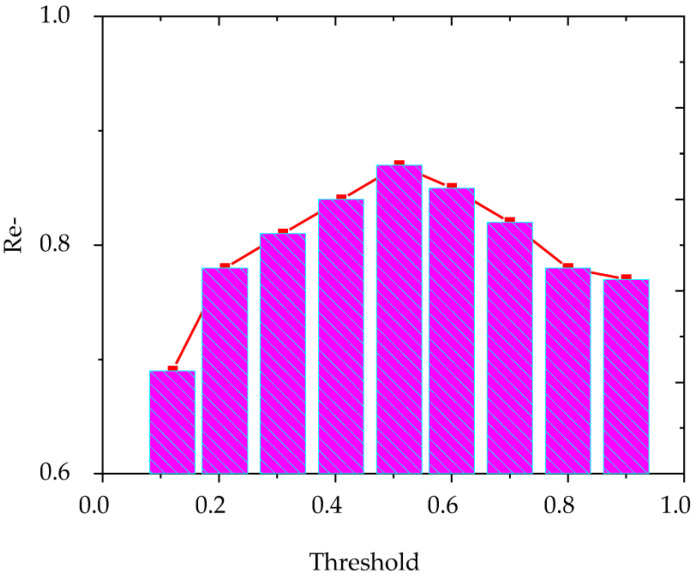
The effect of different thresholds on the regression rate.

The selection of the threshold value in the research algorithm directly affects the clustering effect. Therefore, choosing an appropriate threshold is significant for improving the clustering effect. Here, the review corpus is used as the training set. The recall is adjusted to observe the clustering effect. When the recall value reaches the maximum value, the clustering effect is the best and the value at this time is also used as the threshold. First, product features for Chinese online products are extracted, filtered, and optimized. The similarity between features is calculated using the point mutual information of the equation. Then, the clustering algorithm is performed. The algorithm selects the maximum recall value as the final selection threshold. The selection threshold is revealed in Figure 9. From the figure, when the threshold value is 0.45, the corresponding recall rate is the highest, which is 88.75%. At this time, the corresponding clustering effect is also the best.

Multi-source web reviews are mined through experimental analysis. Table 3 shows the performance comparison results of the PSO-K-means algorithm proposed here and the K-means method only.

Table 3 indicates that using the PSO-K-means algorithm has a higher recall rate than simply using the K-means algorithm. The recall performance of the model represents the proportion of data in the algorithm that is true positive and judged positive. It can be seen that the PSO-K-means algorithm has a promising application in the cluster analysis of related reviews. The main function of PSO is to find the optimal value. The programming implementation of genetic algorithm is relatively complicated and the utilization rate of network feedback information is not high. Therefore, the improved optimization algorithm of K-means + PSO is used here, which has a reference effect on the model experiment of the big data analysis algorithm.

The accuracy values of the PSO-K-means algorithm and the K-means algorithm under different thresholds are further analyzed, as shown in Table 4.

Table 4 compares the accuracy values of the PSO-K-means algorithm and the K-means algorithm. It is found that using the PSO-K-means algorithm has a higher accuracy value than simply using the K-means algorithm and it reaches 90% with a threshold of 0.5. Therefore, the PSO-K-means algorithm has application prospects in the cluster analysis of related reviews. It also has a reference effect on the research of big data analysis algorithms.

Finally, the time required for clustering the PSO-K-means algorithm and the K-means algorithm under different thresholds is analyzed, as shown in Table 5.

Table 5 compares the time required for the PSO-K-means algorithm and the K-means algorithm at different thresholds. It is found that with the threshold increase, the clustering completion time of the PSO-K-means algorithm and the K-means algorithm showed a trend of first decreasing and then rising. Using the PSO-K-means algorithm takes less time than simply using the K-means algorithm and the time required is only 76.32ms at a threshold of 0.8. Therefore, the PSO-K-means algorithm can complete the cluster analysis in a shorter time and the clustering accuracy is better for providing a reference for the research of big data analysis algorithms.

### 4.3. Research on the Impact of the Implementation of International Marine Environment Protection Law on Global Public Health

It is necessary to explore the impact of the implementation of international law on marine environmental protection on global public health. Years of implementation of international law on marine environmental protection increase. The frequency of representative entries related to public health in relevant comments is counted. Among the original 5931 reviews, good reviews containing public health-related terms are preprocessed into 2867. The PSO-K-means algorithm is set to perform year statistics on different time windows; the results are shown in Table 6.

From Table 6, the frequency of public health-related entries in online reviews has increased yearly with the implementation of the international law on marine environmental protection. Especially after 2000, there has been an explosion of commentary on marine environmental protection and public health. This is closely related to the improvement of international marine protection law after 2000. Therefore, implementing marine environmental protection laws is vital in promoting global public health.

### 4.4. Discussion

Gao et al. (2020) studied the application of the PSO algorithm in the K-means algorithm [22]. The PSO-K-means algorithm has good clustering performance and is obviously superior to the existing classical or most advanced clustering algorithms, which is consistent with the research results of Gao et al. (2020) [22]. In summary, the PSO-K-means algorithm can efficiently cluster vulnerability data information. It has the characteristics of high accuracy of the AHC algorithm when applied to the clustering analysis of multi-source online reviews. The development trend of online public opinion information can be judged by adjusting the time window of the algorithm to predict the implementation of the international marine environmental protection law and the global public health image. The results show that the clustering analysis of marine environment by the PSO-K-means algorithm can promote the clustering of marine characteristic words, which has a practical reference value for realizing marine environmental protection and sustainable development of marine resources. Furthermore, through the analysis of the international marine environment, it is found that global warming is one of the possibilities for the deterioration of the international marine environment. As a result, global warming will be mitigated by taking measures such as greening urban spaces and improving the quality of green spaces. This is of great practical significance for establishing healthy communities and can provide concrete measures for the governance of the international marine environment.

## 5. Conclusions

In this paper, the PSO-K-means algorithm is designed and, based on this algorithm, the original marine environmental protection review data set obtained in the network is mined and analyzed in detail. The results show that the designed algorithm has good clustering performance and can promote the clustering of marine feature words, thus promoting the protection of marine environment and the development of global public health communities. Therefore, mitigating global warming by greening urban spaces and improving the quality of green spaces is of great significance to establishing healthy communities and the governance of the international marine environment. The PSO-K-means algorithm applied to the analysis of online review data is more robust than traditional methods, which provides ideas for online review data mining. Through this algorithm, the reactions and opinions of network users can be quickly understood, which affects the judgment of public social opinion. The combined use of the PSO-K-means algorithm is better than K-means alone. After the recall rate test, the recall rate of the PSO-K-means algorithm can reach 88.75%. Therefore, the PSO-K-means algorithm has a good application prospect. However, there are still many details in the process of information processing that are not considered carefully. The solutions to many problems still need to be improved. The effect of data preprocessing to eliminate invalid comments needs to be further strengthened. The visualization of analysis results still needs improvement. This will also be an important research direction for future related work.

## Figures and Tables

**Table 1:** Systematic summary of the literature review data.

Literature Number	Author	Year	Research Content
[15]	Bu et al.	2019	K-means clustering analysis based on leader identification and dynamic game
[16]	Tang	2022	Chinese detection and research of microblog public opinion analysis system
[17]	Rahim Taleqani et al.	2019	Public perception of no-dock bike sharing
[18]	Li et al.	2020	Machine learning intelligent model based on K-means algorithm
[19]	Zheng et al.	2022	Evaluation of small and medium-sized enterprise support policies based on K-means clustering

**Table 2:** Clustering results of marine environmental protection review data.

Number of Clusters	Cluster	Feature Word	Number
K = 6	1	Pollution, marine, and environment	1956
	2	Water quality, resources, and supervision	712
	3	Plastic, leaking, and creature	695
	4	Life, ship, and development	679
	5	Food chain, health, and transportation	476
	6	Legislation, ocean currents, and red tides	312
K = 5	1	Pollution, marine, and environment	2214
	2	Water quality, resources, and supervision	806
	3	Plastic, leaking, and creature	714
	4	Life, ship, and development	695
	5	Food chain, health, and transportation	401
K = 4	1	Pollution, marine, and environment	2367
	2	Water quality, resources, and supervision	984
	3	Plastic, leaking, and creature	758
	4	Life, ship, and development	721

**Table 3:** Comparison of recall rates of two algorithms.

Threshold	Recall	
	PSO-K-Means	K-Means
0.1	0.68	0.68
0.2	0.77	0.76
0.3	0.80	0.79
0.4	0.82	0.81
0.5	0.85	0.83
0.6	0.83	0.83
0.7	0.82	0.81
0.8	0.77	0.77
0.9	0.77	0.75

**Table 4:** Comparative analysis table of accuracy values of the two algorithms.

Accuracy	Threshold								
	0.1	0.2	0.3	0.4	0.5	0.6	0.7	0.8	0.9
PSO-K-means	0.72	0.81	0.85	0.86	0.90	0.88	0.86	0.81	0.81
K-means	0.72	0.80	0.83	0.85	0.87	0.86	0.86	0.81	0.80

**Table 5:** Comparative analysis table of the time required for clustering of the two algorithms.

Time (ms)	Threshold								
	0.1	0.2	0.3	0.4	0.5	0.6	0.7	0.8	0.9
PSO-K-means	88.72	84.11	82.03	78.44	77.99	77.72	76.99	76.32	77.62
K-means	89.12	85.01	83.55	81.81	80.13	80.14	78.96	79.02	81.84

**Table 6:** Statistics of comment information in different time windows.

Feature Word	Years	Word Frequency
Health	1982–1992	3
Pollution	1992–2000	16
Environment	2000–2010	491
Legislation	2010–2021	2357
Feature word	Years	Word frequency

## Data Availability

Not applicable.
